# Plasma Urea Cycle Metabolites May Be Useful Biomarkers in Children With Eosinophilic Esophagitis

**DOI:** 10.3389/fped.2018.00423

**Published:** 2019-01-10

**Authors:** Lindsay M. Moye, Yuying Liu, Cristian Coarfa, Nagireddy Putluri, Jon Marc Rhoads

**Affiliations:** ^1^Department of Pediatric Gastroenterology, University of Texas McGovern Medical School, Houston, TX, United States; ^2^Department of Molecular and Cellular Biology, Advanced Technology Core, Baylor College of Medicine, Houston, TX, United States

**Keywords:** eosinophilic esophagitis, metabolomics, biomarker, discovery, dimethylarginine, putrescine

## Abstract

**Background:** Eosinophilic esophagitis (EoE) is a disorder of the esophagus that has become increasingly recognized in children. Because these children undergo multiple endoscopies, discovering a non-invasive biomarker of disease activity is highly desirable. The aim of this study was to use targeted plasma metabolomics to identify potential biomarker candidates for EoE in a discovery phase.

**Methods:** A prospective, single-center clinical trial was performed on 24 children ages 2–18 years with and without EoE undergoing upper endoscopy for any indication. Blood samples were collected for metabolomics profiling using the subclasses: amino acids, tricarboxylic acid cycle, acetylation, and methylation. Using mass spectrometry and systematic bioinformatics analysis, 48 metabolites were measured and compared between children with active EoE (+EoE) and controls (–EoE). To investigate the effect of proton pump inhibitor (PPI) use on metabolites, patients were also stratified based on PPI use (+PPI, –PPI).

**Results:** Seven children had active EoE at the time of endoscopy. Eleven children were on PPI (4 with EoE). Of the 48 metabolites measured, 8 plasma metabolites showed statistically significant differences (*p* < 0.05) comparing +EoE –PPI to –EoE –PPI, a few of which were upregulated metabolites involved in the urea cycle. There were 14 significant differences comparing +EoE +PPI to +EoE –PPI. This demonstrated that in EoE patients, PPI use upregulated metabolites involved in the urea cycle, while it downregulated metabolites involved in methylation. Comparison among all four groups, +EoE +PPI, +EoE –PPI, –EoE +PPI, and –EoE –PPI, revealed 27 significantly different metabolites. +EoE +PPI had downregulated methionine and N-acetyl methionine, while both +EoE groups and –EoE +PPI had upregulated homocysteine, N-acetylputrescine, N-acetylornithine, arginine, and ornithine.

**Conclusion:** The present study revealed key plasma metabolite differences in children with EoE compared to unaffected controls. Notable candidate biomarkers include dimethylarginine, putrescine, and N-acetylputrescine. PPI use was shown to influence these urea cycle metabolites, regardless of EoE presence. Therefore, future studies should distinguish patients based on PPI use or determine metabolites while not on treatment. These findings will be confirmed in a larger validation phase, as this may represent a significant discovery in the search for a non-invasive biomarker for EoE.

**Clinical Trial Registration:** This clinical trial was registered with ClinicalTrials.gov, identifier: NCT 03107819.

## Introduction

Eosinophilic esophagitis (EoE) is a clinicopathologic disorder of the esophagus requiring esophageal dysfunction and one mucosal biopsy containing ≥15 eosinophils (eos) per high-power field (hpf) for diagnosis (1). Additionally, eosinophil-predominant inflammation must persist after an 8 week trial of proton-pump inhibitor (PPI) therapy (2) to exclude PPI-responsive esophageal eosinophilia (3).

EoE has become increasingly recognized in children with an incidence of ~1:10,000 (4). Clinical presentation varies greatly and depends on age (5). Presenting symptoms may include feeding difficulties, failure to thrive and vomiting in early childhood, and in adolescents and adults, abdominal pain, heartburn, chest pain, difficulty swallowing and food impaction (1, 6).

The pathophysiology of EoE is not fully understood but it is thought to be a delayed hypersensitivity that is T helper cell type 2 predominant and interleukin 4 (IL-4), IL-5, and IL-13 dependent (6, 7). EoE is associated with food antigen-driven hypersensitivity, as evidenced by its response to dietary therapy.

Treatment is based on control of inflammation either with topical corticosteroids or food-antigen avoidance. Swallowed steroids are effective; however, when discontinued, the disease almost always recurs (2). Side effects from the long-term use of steroids in children remains a concern (3), so dietary therapy has been strongly recommended (2). Various food elimination diets have been proposed which focus on identifying food triggers based on the absence of clinical symptoms and histological remission of esophageal eosinophilia. This process is laborious and difficult for the patient as it requires upper endoscopy before and after dietary changes are made to verify favorable histological response, often multiple times in a year when trying to identify causative food(s).

Following diagnosis, the absence or presence of symptoms does not reliably correspond to disease activity (1). Some experts recommend periodic surveillance even in the absence of symptoms due to long-term sequelae of untreated inflammation (8). Untreated EoE can progress to esophageal fibrosis and dysfunction (6), and eventually stricture and food impaction (3).

Therefore, identification of a non-invasive biomarker for measuring disease activity is highly desirable, not just for making the initial diagnosis, but also for monitoring response to therapy. Previous research has examined peripheral blood eosinophils, a multitude of cytokines and chemokines, eosinophil-derived neurotoxin, eotaxin-3, and many others, but none have been proven adequate for non-invasive monitoring (7). To our knowledge, the utilization of metabolomics has not been performed in children or adults with EoE.

Metabolomics is a high-throughput technique for directly measuring biochemical activity by monitoring the substrates and products transformed during cellular metabolism (9). Through the use of mass spectrometry, it offers the unique opportunity to rapidly measure thousands of metabolites simultaneously from only minimal amounts of biological sample (9).

In an effort to identify biomarkers for EoE, we performed targeted plasma metabolomics on EoE patients and compared them to controls. We then further distinguished them based on PPI use.

## Materials and Methods

### Study Population

Patients were prospectively enrolled at Children's Memorial Hermann Hospital in Houston, Texas from March 2017 to March 2018. Eligible patients were between the ages of 2 and 18 years, with and without known EoE, undergoing upper gastrointestinal endoscopy for any indication. Patients were excluded if they had a known diagnosis of another disorder with similar clinical, histological or endoscopic features of EoE, such as PPI-responsive esophageal eosinophilia, Crohn's disease, infectious esophagitis, drug-associated esophagitis, collagen vascular disease, hypereosinophilic syndromes, or eosinophilic gastroenteritis. A child already known to have EoE was excluded if he or she had a biopsy demonstrating <15 eos/hpf at the time of the study. This study was carried out in accordance with the recommendations of the University of Texas's Institutional Review Board, with written informed consent from the parent or guardian of all subjects. All subjects gave written informed consent in accordance with the Declaration of Helsinki. The protocol was approved by the University of Texas's Institutional Review Board, HSC-MS-16-1078.

### Sample Collection and Endoscopy

Ten to 15 milliliters of peripheral blood was collected at the time of IV placement. The blood was then aliquoted into vials and sent for complete blood count with differential, comprehensive metabolic panel, and plasma metabolomics processing.

EGD was performed in all patients with the use of standard Olympus instruments by pediatric gastroenterologists. Biopsy specimens were obtained with cold forceps at the discretion of the endoscopist from at least two levels of the esophagus and immediately placed in formalin and labeled by location. Sections were cut from routinely processed formalin-fixed paraffin-embedded tissue blocks and were stained with hematoxylin and eosin. Slides were examined in random order by one of two pathologists blinded to the fact that the patient was enrolled in a research study. The peak eosinophil count for each biopsy specimen was defined as the maximum number of eosinophils in any single high power field.

Blood samples for complete blood count with differential and comprehensive metabolic panel were collected in the appropriate tubes and immediately processed. Blood samples for biomarker measurements were collected in heparinized tubes, and plasma was extracted and stored at −80 degrees until batch submission for processing.

### Metabolomics

Using mass spectrometry, 48 metabolites were measured and compared between children with active EoE and unaffected controls.

Metabolites and internal standards, including N-acetyl aspartic acid-d3, [15N]2 Tryptophan, Glutamic acid-d5, Thymine-d4, Gibberellic acid, Trans-Zeatine, Jasmonic acid, [15N] Anthranilic acid, and Testosterone-d3, were purchased from Sigma-Aldrich (St. Louis, MO).

### Extraction

100 ul of plasma was used for extraction. The extraction step started with 750 μL ice-cold methanol:water (4:1) containing 20 μL spiked internal standards was added to each test and quality control samples. Ice-cold chloroform and water was added in a 3:1 ratio for a final proportion of 1:4:3:1 water:methanol:chloroform:water. The organic (methanol and chloroform) and aqueous layers were dried and re-suspended with 50:50 methanol:water. The extract was deproteinized using a 3-kDa molecular filter (Amicon Ultracel-3K Membrane; Millipore Corporation, Billerica, MA) and the filtrate was dried under vacuum (Genevac EZ-2plus; Gardiner, Stone Ridge, NY). Prior to mass spectrometry, the dried extracts were re-suspended in identical volumes of solvent composed of 1:1 water:methanol and subjected to liquid chromatography-mass spectrometry 10 μL of suspended samples was injected and analyzed using a 6490 triple quadrupole mass spectrometer (Agilent Technologies, Santa Clara, CA) coupled to a HPLC system (Agilent Technologies, Santa Clara, CA) via single reaction monitoring.

### Measurement of Amino Acids

ESI positive mode was used in method. The HPLC column was Zorbax eclipse XDB C-18, 1.8 micron, 4.6 × 100 mm. Mobile phase A and B were 0.1% formic acid in water and acetonitrile, respectively. Gradient: 0 min-2% B; 6 min- 2% of B, 6.5 min-30% B, 7 min- 90% of B, 12 min 95% of B, 13 min 2% of B followed by re-equilibration at end of the gradient, 20 min to the initial starting condition 2% of B. Flow rate: 0.2 ml/min.

### Measurement of TCA Cycle

ESI negative mode was used in method. The HPLC column was Luna 3μM NH2 100A^0^, 150 × 2 mm. Mobile phase A and B were 5 mM ammonium acetate in water and 100% acetonitrile, respectively. Gradient: 0 min-80% B (0.2 ml/min); 20 min-2% of B (0.2 ml/min), 20.1 min-2 % B (0.3 ml/min), 25 min-80% B (0.3 ml/min), 30 min 80% of B (0.35 ml/min), 35 min 80% of B (0.4 ml/min), 37.8 min 80% of B (0.4 ml/min), 37.99 min 80% B (0.2 ml), followed by re-equilibration end of the gradient 37.99 min to the initial starting condition 80% B.

### Measurements of Methylated and Acetylated Metabolites

We used two methods as described below to separate the metabolites:

Method A: ESI positive mode was used in method A. The HPLC column was Waters Xbridge Amide 3.5 μm, 4.6 × 100 mm. Mobile phase A and B were 0.1% formic acid in water and acetonitrile, respectively. Gradient: 0 min-85% B; 3–12 min-85 to 30% B, 12–15 min-2% B, 16 min-95% -B, followed by re-equilibration end of the gradient, 23 min to the initial starting condition 85% B. Flow rate: 0.3 ml/min. Injection volume 5 ul.

Method B: ESI negative mode was used in method B. The HPLC column was Waters Xbridge Amide 3.5 μm, 4.6 × 100 mm. Mobile phase A and B were 20 mM ammonium acetate in water with pH 9.0 and 100% acetonitrile, respectively. Gradient: 0 min-85 % B; 0–3 min-85 to 30% B, 3–12 min-30%-2% B, 12–15 min-2% -B, 15–16 min-85% B followed by re-equilibration end of the gradient- the 23 min to the initial starting condition 85% B. Flow rate: 0.3 ml/min. Injection volume 5 ul.

### Analysis

The data was log2-transformed and normalized with internal standards on a per-sample, per-method basis. For every metabolite in the normalized dataset, two sample *t*-tests were conducted to compare expression levels between Ta and other stages (T1-T4). Differential metabolites were identified by adjusting the *p*-values for multiple testing at an FDR threshold of <0.25. A hierarchical cluster of the differentially expressed metabolites was generated using the R statistical software system (https://www.r-project.org/).

## Results

### Patient Characteristics

Twenty-four patients were enrolled in the study (Table 1). Seven had active EoE at the time of endoscopy. The remaining 17 participants had a normal EGD, both grossly and histologically. Two patients did not have an adequate blood specimen to send for complete blood count with differential. In one instance, the patient had received IV midazolam just prior to specimen collection. All patients were verified to have a normal comprehensive metabolic panel as a screening method to detect potential renal or hepatic dysfunction.

**Table 1:** Characteristics of children with EoE and unaffected controls.

**Patient**	**Age (*y*)**	**Sex**	**Ethnicity**	**Race**	**PPI**	**Topical Steroid**	**Peak eos count per level (P, M, D)**	**Atopic Phenotype**	**Indication for EGD**	**Other Diagnoses**
**ACTIVE EOE (*****n*** **=** **7)**										
3	12	M	NH	C	+	+	10, 22, 30	+E, +AR	V, Dys, EoE	ADHD
12	9	M	H	C	+	+	15, 22, 45	+AR	FTT	±
15	7	M	H	C		+	40, 50, 60	+A	EoE, FD	
18	5	F	H	C			12, >40, >40		FTT	
20	12	M	NH	AA	+		30, >50, >50	+AR, +A	EoE	ASD, Sz
22	11	M	NH	C			>30, >30, >60	+E, +A, +AR	EoE, V	ADHD
24	9	M	NH	C	+	+	>100, >60, >100	+E, +AR	EoE	ADHD
**UNAFFECTED CONTROLS (*****n*** **=** **17)**										
1	12	F	NH	C			0, 0, 0	+AR, +A	V, Dys, Abd pain	
2^*^	14	F	NH	AA			0, 0, 0		Abd pain	ADHD, ODD
4^*^	10	M	H	C	+		0, 0, 0	+AR	Dys	
5	13	M	H	C			0, 0, 0	+AR, +A	Wt loss	
6	5	F	NH	C			0, 0, 0	+AR	CP	
7	13	M	NH	C	+		0, 0, 10	+AR, +A	GERD, V, Abd pain	CDH
8	8	F	NH	C	+		0, 0, 0		CP, Abd pain	Co
9	13	F	H	C	+		0, 0, 0	+AR, +A	Abd pain, V	ADHD
10	6	F	NH	A			N/A, 0, 0	+AR	Abd pain	
11^†^	15	F	NH	C			0, 0, 4	+AR	GERD	ADHD
13	9	M	NH	C	+		N/A, 0, 0		GERD, Dys	
14	8	M	NH	E			0, 0, 0		Abd pain	
16	7	F	NH	AA			0, 0, 0	+AR	Abd pain	ADHD
17	13	F	NH	C			0, 0, 0	+E, +AR	Abd pain	
19	12	M	NH	C-I			0, 0, 0	+AR	Poor wt gain	ADHD, SS
21	8	M	Unk	C	+		0, 0, 5	+AR, +A	GERD, Dys	
23	2	F	NH	A	+		0, 0, 0	+AR	GERD, Dys, V	

*P, proximal; M, middle; D, distal; EGD, esophagogastroduodenoscopy; NH, non-Hispanic; H, Hispanic; Unk, unknown; C, Caucasian; AA, African-American; E, Egyptian; C-I, Caucasian-Indian; E, eczema; AR, allergic rhinitis; A, asthma; V, vomiting; Dys, dysphagia; EoE, eosinophilic esophagitis; FTT, failure to thrive; FD, feeding difficulties; Abd, abdominal; Wt, weight; CP, chest pain; GERD, gastroesophageal reflux disease; ADHD, attention deficit hyperactivity disorder; ASD, autism spectrum disorder; Sz, seizure disorder; ODD, oppositional defiant disorder; CDH, congenital diaphragmatic hernia; Co, constipation; SS, short stature*.

*± Jejunal atresia with short gut syndrome and liver failure status post liver, small bowel and pancreas transplant*.

* *No complete blood count*.

† *Received IV midazolam prior to specimen collection*.

### Plasma Metabolomics

**Figure 1:**
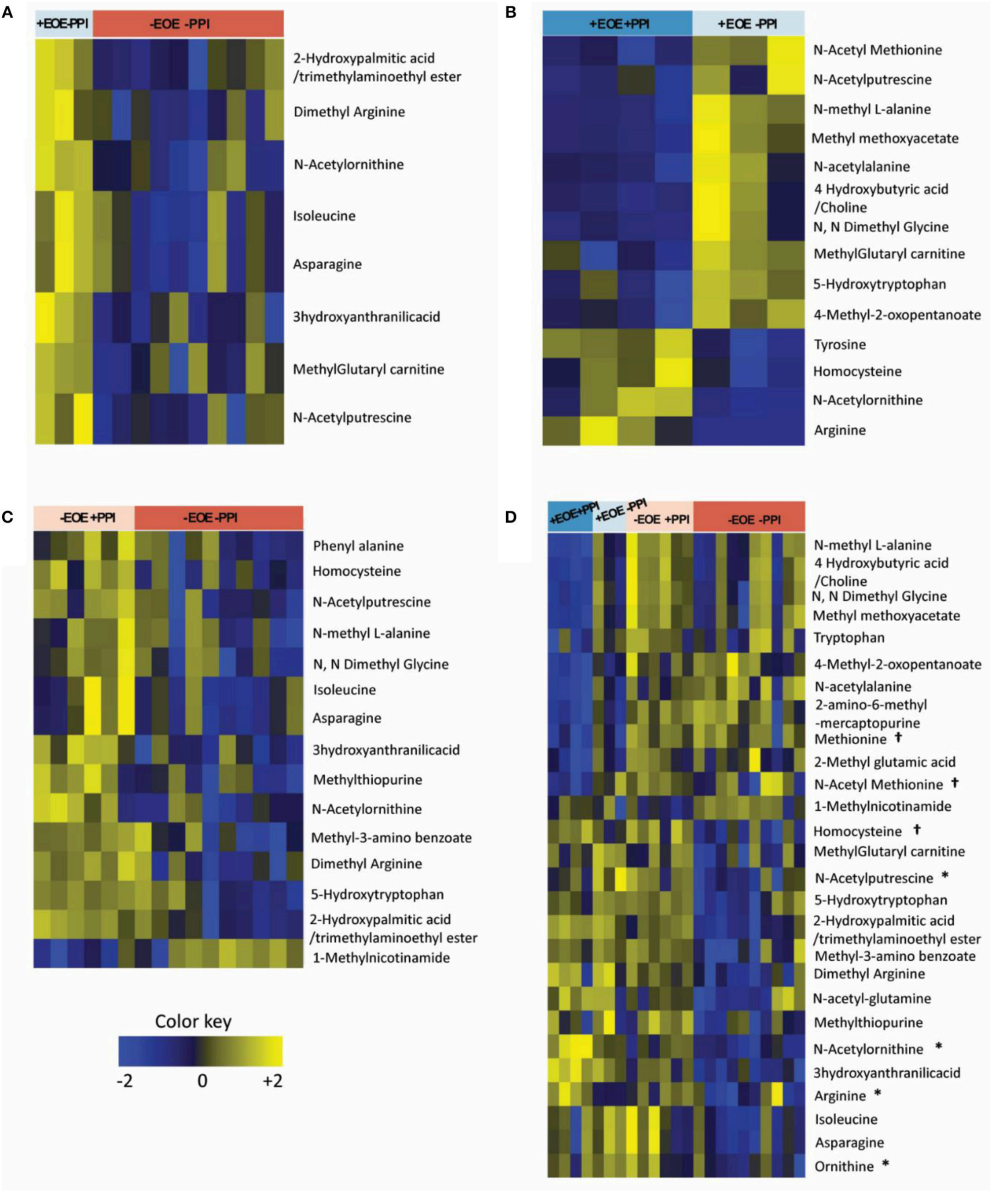
Heat maps representing the differentially altered metabolites (FDR <0.25). Upregulated metabolites are shown in yellow, downregulated metabolites in blue, and those with no changes in black. **(A)** Children with EoE, not on PPI, compared to children without EoE, not on PPI. **(B)** Children with EoE, on PPI, compared to children with EoE, not on PPI. **(C)** Children without EoE, on PPI, compared to children without EoE, not on PPI. **(D)** Comparison of all 4 groups. ^†^Metabolites involved in methionine methylation. ^*^Metabolites involved in the urea cycle.

Of the 48 metabolites measured, 8 plasma metabolites showed statistically significant differences (*p* < 0.05) comparing +EoE –PPI to –EoE –PPI. On the heat maps, upregulated metabolites are shown in yellow, downregulated metabolites in blue, and those with no changes in black (Figure 1A). Our findings showed that in EoE patients, EoE upregulated metabolites were mainly involved in the urea cycle. Dimethyl arginine was increased in all children with EoE but was also increased in those without EoE who were taking PPIs. N-acetyl putrescine was elevated in 3 of 4 children with EoE not taking PPIs and in all 3 of those with EoE who were taking PPIs; however it was also elevated in non-EoE children taking PPIs. N-acetyl ornithine was elevated strongly in 3 of 4 +EoE –PPI and mildly elevated in +EoE+PPI, but this metabolite was only mildly elevated and only in 4 of 17 –EoE –PPI children.

We next examined the effects of PPI treatment on plasma metabolites in children with EoE. There were 14 significant differences comparing +EoE +PPI to +EoE –PPI (Figure 1B). This demonstrated that in EoE patients, PPI use upregulated metabolites involved in the urea cycle, while it downregulated metabolites involved in methylation.

We also examined the effects of PPI treatment on plasma metabolites in children without EoE. There were 15 significantly altered metabolites comparing –EoE +PPI to –EoE –PPI (Figure 1C). In these non-EoE patients, PPI use upregulated metabolites involved in both methylation and the urea cycle.

**Figure 2:**
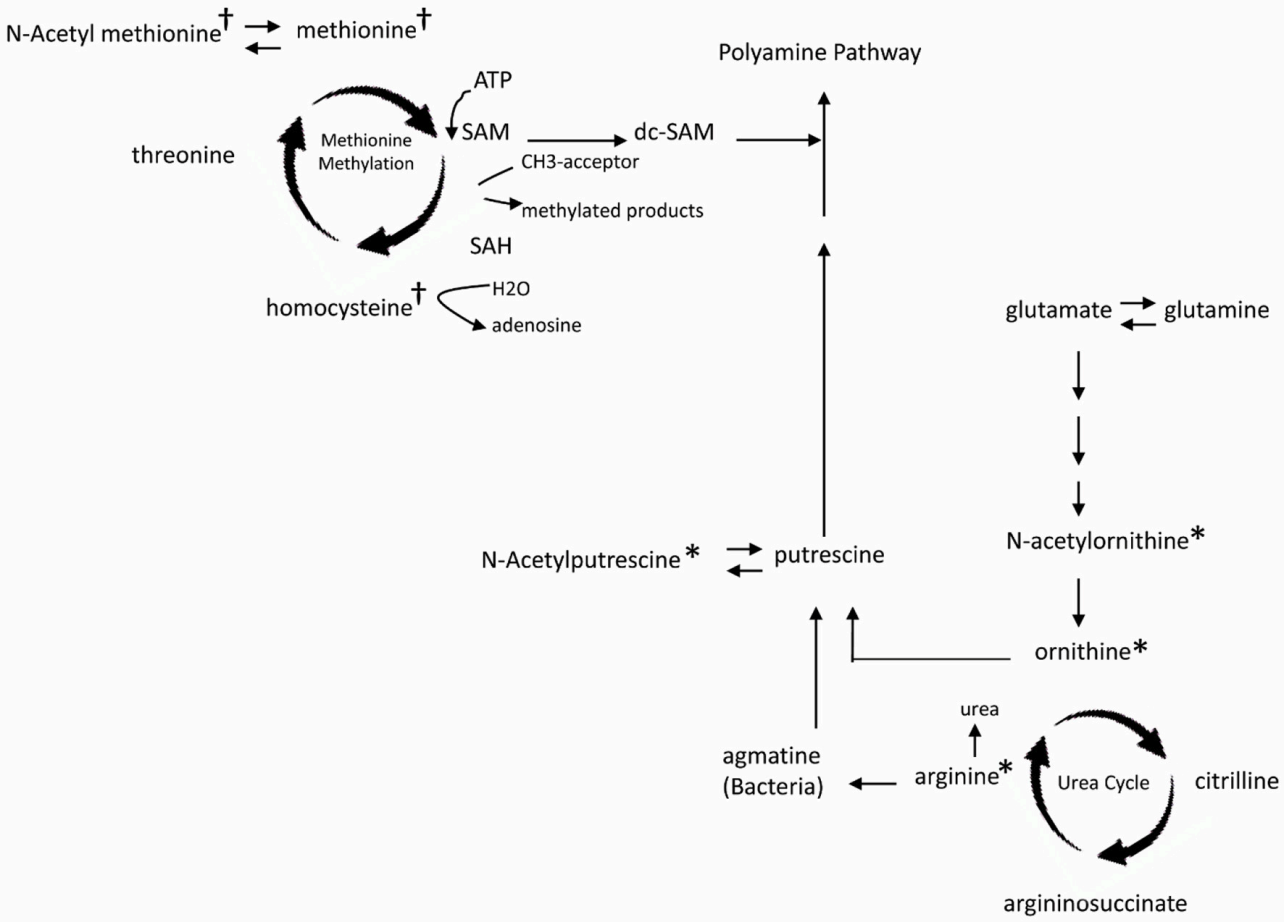
Major changes of plasma metabolites in children with EoE and PPI treatment related to metabolism of methionine methylation (†) and urea cycle (^*^). SAM, S-adenosyl methionine; dc-SAM, decarboxylated SAM; SAH, S-adenosyl homocysteine.

Comparison among all 4 groups (+EoE +PPI, +EoE –PPI, –EoE +PPI and –EoE –PPI) revealed 27 significantly different metabolites (Figure 1D). Figure 2 highlights some of these metabolites' roles in methionine methylation and the urea cycle. +EoE +PPI had downregulated methionine and N-acetyl methionine, while both +EoE groups and –EoE +PPI had upregulated homocysteine, N-acetylputrescine, N-acetylornithine, arginine, and ornithine.

**Figure 3:**
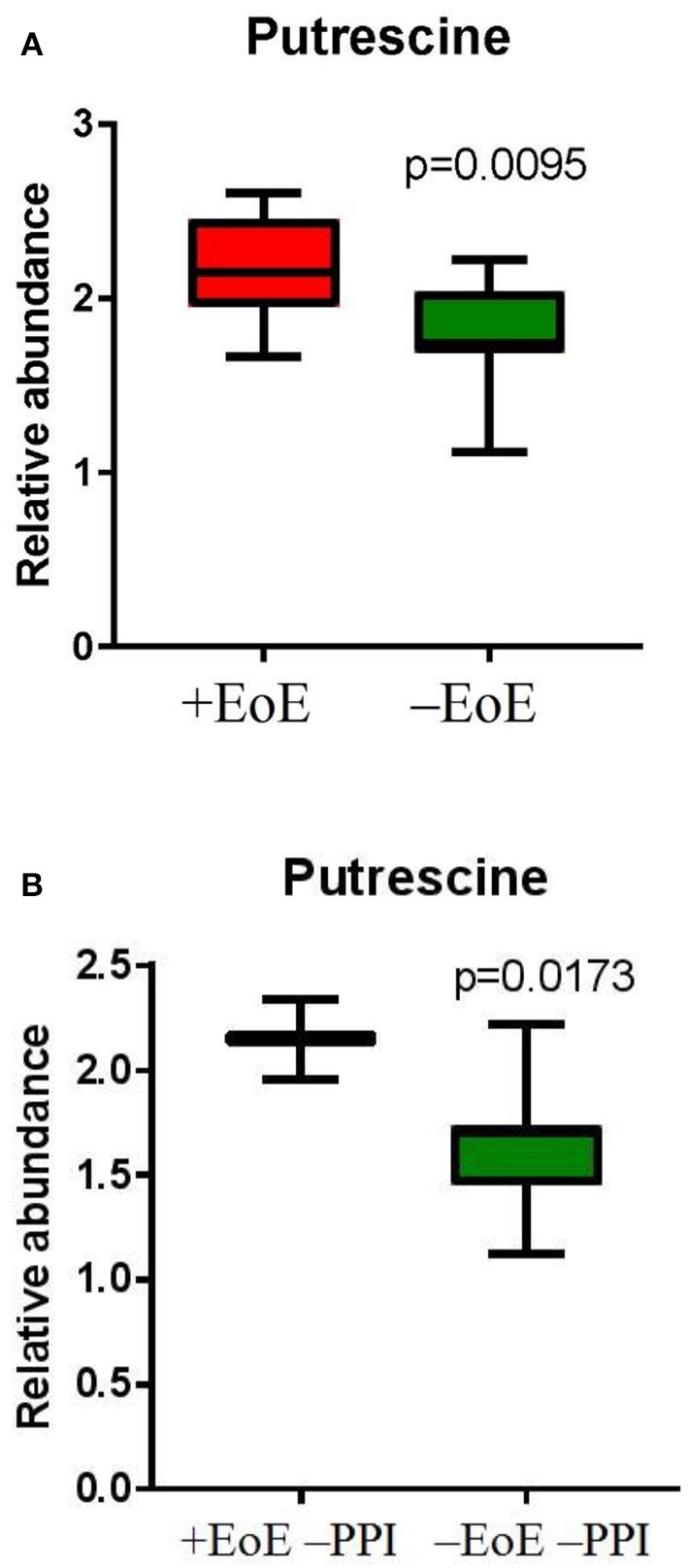
Relative abundance of putrescine in **(A)** EoE children compared with unaffected controls; and **(B)** EoE children, not on PPI compared to non-EoE children, not on PPI.

To further evaluate putrescine, a key metabolite in the urea cycle, we measured the relative abundance of putrescine and compared the EoE groups to those without EoE and found putrescine to be significantly elevated in children with EoE (*p* = 0.009, Figure 3A). We also wanted to see if putrescine was increased in the disease group compared with controls in the absence of PPI; therefore, we measured the relative abundance of putrescine in +EoE –PPI vs. –EoE –PPI. We found it was significantly increased in EoE children not taking PPI compared to non-EoE children not taking PPI (*p* = 0.017, Figure 3B).

## Discussion

Although endoscopy with biopsy provides an objective assessment of EoE activity, it is costly, burdensome to patients, and carries the risk of serious complications (10). The discovery and qualification of a non-invasive biomarker for EoE would help resolve some of these issues. Plasma would be an ideal biofluid because it can be easily collected in a pediatric patient in a clinic, hospital or laboratory setting. Ultimately, the goal for the clinician is to be able to use a non-invasive test to aid in diagnosing EoE and monitoring response to treatment with dietary changes or topical steroids, especially since symptom reporting can be challenging for children, and symptoms are not always reliable indicators of disease severity.

The present study evaluated plasma metabolites, and demonstrated, for the first time, key metabolite differences in children with EoE compared to unaffected controls. Interestingly, there were also distinct differences in metabolite concentrations depending on PPI use.

### Dimethylarginine

Of note, we found dimethylarginine to be upregulated in children with EoE (regardless of PPI use) and it was also increased in non-EoE children taking PPI. Dimethylarginine is a uremic toxin that inhibits nitric oxide (NO) production by competing with L-arginine at the active site of NO synthase and can further be classified as symmetric or asymmetric (11). Our study did not distinguish between these two stereoisomers, but this can be done using HPLC. Elevations of both asymmetric dimethylarginine (ADMA) and symmetric dimethylarginine (SDMA) have been shown to play multifunctional roles in many human diseases (12) and are implicated in promoting endothelial injury (11). High levels of ADMA have been associated with cardiovascular disease (11), chronic kidney disease (13), and poor outcomes in asthma (14). Similarly, ADMA and SDMA levels have been shown to be significantly elevated in patients with inflammatory bowel disease (15), and chronic lymphocytic leukemia (16). These studies collectively suggest that high dimethylarginine levels are associated with poor outcomes in various chronic and inflammatory conditions. Thus, it makes sense for dimethylarginine to be increased in the plasma of children with an inflammatory condition like EoE. Perhaps the mechanism in EoE is that this endothelial dysfunction leads to enhanced blood cell (eosinophil) migration and increased local chemokine and/or cytokine release at the esophageal level.

The elevation of dimethylarginine in non-EoE children on PPI can be explained by previous research demonstrating that PPIs increase ADMA levels because they bind to and inhibit dimethylarginine dimethylaminohydrolase, the enzyme that degrades ADMA (17). Dimethylarginine levels (or, more specifically, ADMA or SDMA levels) should be further examined as they may reflect EoE disease activity. This is especially of interest because previous research has demonstrated that the effects of ADMA can be counteracted by administration of the NO precursor, L-arginine, likely via a direct competition for the NO synthase enzyme (18). Thus, L-arginine could serve as a novel therapeutic agent for EoE.

### Putrescine

Perhaps even more important, we found putrescine to be significantly increased in EoE children compared to controls, as well as in EoE children not on PPI compared to controls not on PPI. Putrescine is a toxic polyamine that is produced during tissue decomposition by the decarboxylation of the urea cycle byproducts arginine and ornithine (19). Putrescine has been shown to contribute to the putrid odor of conditions such as halitosis (bad breath) and bacterial vaginosis (20). We hypothesize that there may be a relationship between cell damage and turnover in EoE and increased levels of putrescine.

Current developments in metabolic profiling technologies have led to the emergence of rapid analysis of thousands of molecules concurrently. However, understanding these unique metabolic fingerprints and how they apply to disease pathophysiology remains a challenge. Our study has several weaknesses, including small sample size, and we did not compare children with other types of esophagitis, such as fungal, peptic, or caustic esophagitis. It is our opinion that the main need for a marker is to follow the course of already established EoE; that is, the initial diagnosis of this chronic disease will not be replaced by finding a metabolic signature.

Our findings represent an important step in the development of more efficient and less invasive diagnostic and disease-monitoring methods in EoE but future research will need to investigate these candidate biomarkers in a validation phase and better understand their role in the various metabolic pathways. Additionally, the present study revealed the importance in distinguishing patients based on PPI use because it appears that PPI use influences urea cycle-related metabolites. Thus, the optimal way to measure urea cycle derivatives in patients with EoE may be to determine their levels when the child is not taking PPI.

### Conclusions

Specific metabolites of the urea cycle related to methionine, ornithine, and polyamine metabolism (especially putrescine) are elevated in children with EoE, but they are modified by PPI treatment. These metabolites may originate from tissue destruction and remodeling. Further research should seek to validate these findings using sequential determination of one or more of these markers, determining reproducibility, variability, and specificity as potential non-invasive markers during the evolution of EoE.

## Data Availability Statement

All relevant data is contained within the manuscript.

## Author Contributions

LM, NP, and JR all contributed to concept development, writing and review of this manuscript. YL contributed to writing and review. CC contributed to data analysis. All authors provided final approval of the version to be published.

### Conflict of Interest Statement

The authors declare that the research was conducted in the absence of any commercial or financial relationships that could be construed as a potential conflict of interest.

## References

[1] Dellon ES Gonsalves N Hirano I Furuta GT Liacouras CA Katzka DA. ACG clinical guideline: evidenced based approach to the diagnosis and management of esophageal eosinophilia and eosinophilic esophagitis (EoE). Am J Gastroenterol. (2013) 108:679–92. 10.1038/ajg.2013.71 23567357

[2] Liacouras CA Furuta GT Hirano I Atkins D Attwood SE Bonis PA. Eosinophilic esophagitis: updated consensus recommendations for children and adults. J Allergy Clin Immunol. (2011) 128:3–20. 10.1016/j.jaci.2011.02.040 21477849

[3] Cianferoni A Spergel JM. Immunotherapeutic approaches for the treatment of eosinophilic esophagitis. Immunotherapy (2014) 6:321–31. 10.2217/imt.14.3 24762076 PMC4697927

[4] Moawad FJ. Eosinophilic esophagitis: incidence and prevalence. Gastrointest Endosc Clin N Am. (2018) 28:15–25. 10.1016/j.giec.2017.07.001 29129296

[5] Markowitz JE Clayton SB. Eosinophilic esophagitis in children and adults. Gastrointest Endosc Clin N Am. (2018) 28:59–75. 10.1016/j.giec.2017.07.004 29129300

[6] Davis BP Pathophysiology of eosinophilic esophagitis. Clin Rev Allerg Immunol. (2018) 154:333–45. 10.1007/s12016-017-8665-9 29332138

[7] Gupta SK. Noninvasive markers of eosinophilic esophagitis. Gastrointest Endosc Clin N Am. (2008) 18:157–67. 10.1016/j.giec.2007.09.004 18061109

[8] Furuta GT Liacouras CA Collins MH Gupta SK Justinich C Putnam PE. Eosinophilic esophagitis in children and adults: a systematic review and consensus recommendations for diagnosis and treatment. Gastroenterol (2007) 133:1342–63. 10.1053/j.gastro.2007.08.017 17919504

[9] Patti GJ Yanes O Siuzdak G. Metabolomics: the apogee of the omics trilogy. Nature (2012) 13:263–9. 10.1038/nrm3314 22436749 PMC3682684

[10] Khanna R Narula N Feagan BG. The role of biomarkers in clinical trials of inflammatory bowel disease. Inflamm Bowel Dis. (2018) 24:1619–23. 10.1093/ibd/izy195 29846593

[11] Dimitroulas T Hodson J Sandoo A Smith J Kitas GD. Endothelial injury in rheumatoid arthritis: a crosstalk between dimethylarginines and systemic inflammation. Arthritis Res Ther. (2017) 19:32. 10.1186/s13075-017-1232-1 28183353 PMC5301328

[12] Tain Y-L Hsu C-N. Toxic dimethylarginines: asymmetric dimethylarginine (ADMA) and symmetric dimethylarginine (SDMA). Toxins (2017) 9:92. 10.3390/toxins9030092 28272322 PMC5371847

[13] Krzanowski M Krzanowska K Gajda M Dumnicka P Kopeć G Guzik B. Asymmetric dimethylarginine as a useful risk marker of radial artery calcification in patients with advanced kidney disease. Pol Arch Intern Med. (2018) 128:157–65. 10.20452/pamw.4201 29600966

[14] Tajti G Papp C Kardos L Keki S Pak K Szilasi ME. Positive correlation of airway resistance and serum assymetric dimethylarginine (ADMA) in bronchial asthma patients lacking evidence for systemic inflammation. Aller Asthma Clin Immunol. (2018) 14:2. 10.1186/s13223-017-0226-5 29308071 PMC5751874

[15] Owczarek D Cibor D Mach T. Asymmetric dimethylarginine (ADMA), symmetric dimethylarginine (SDMA), arginine, and 8-iso-prostaglandin F2alpha (8-iso-PGF2alpha) level in patients with inflammatory bowel diseases. Inflamm Bowel Dis. (2010) 16:52–7. 10.1002/ibd.20994 19575355

[16] Chachaj A Wiśniewski J Rybka J Butrym A Biedron M Krzystek-Korpacka M. Asymmetric and symmetric dimethylarginines and mortality in patients with hematological malignancies- a prospective study. PLoS ONE (2018) 13:e0197148. 10.137/journal.pone.0197148 29787597 PMC5963779

[17] Ghebremariam YT LePendu P Lee JC Erlanson DA Slaviero A Shah NH. Unexpected effect of proton pump inhibitors: elevation of the cardiovascular risk factor asymmetric dimethylarginine. Circulation (2013) 128:845–53. 10.1161/circulationaha.113.003602 23825361 PMC3838201

[18] Böger RH Bode-Böger SM Szuba A Tsao PS Chan JR Tangphao O. Asymmetric dimethylarginine (ADMA): a novel risk factor for endothelial dysfunction. Circulation (1998) 98:1842–7. 10.1161/01.cir.98.18.1842 9799202

[19] Wisman A Shrira I. The smell of death: evidence that putrescine elicits threat management mechanisms. Front Psychol. (2018) 6:1274. 26379597 10.3389/fpsyg.2015.01274 PMC4551835

[20] National Center for Biotechnology Information PubChem Compound Database; CID = 1045. (2018). Available online at: https://pubchem.ncbi.nlm.nih.gov/compound/1045 (Accessed July 12, 2018).

